# Growth Inhibition by Caffeic Acid, One of the Phenolic Constituents of Honey, in HCT 15 Colon Cancer Cells

**DOI:** 10.1100/2012/372345

**Published:** 2012-04-29

**Authors:** Saravana Kumar Jaganathan

**Affiliations:** Department of Biomedical Engineering, PSNA College of Engineering and Technology, Dindugal, Kothandaraman Nagar, Dindugal 624622, Tamil Nadu, India

## Abstract

Previous work from our laboratory showed that the mechanism of crude-honey induced apoptosis in colon cancer cells. Since phenolic constituents of honey were attributed to its apoptosis-inducing ability, we studied caffeic acid, one of the phenolic constituents of honey, induced effect on colon cancer cells. Antiproliferative effect of caffeic acid was estimated using 3-(4,5-dimethylthiazol-2-yl)-2,5-diphenyl tetrazolium bromide (MTT) assay. MTT assay signified the antiproliferative nature of caffeic acid against the HCT 15 colon cancer cells. A time-dependent inhibition of colony formation was evident with caffeic acid treatment. Cell-cycle analysis of caffeic acid- (CA-) treated cells indicated increasing accumulation of cells at sub-G_1_ phase. Photomicrograph images of treated cells showed membrane blebbing and cell shrinkage. Yo-pro-1 staining of caffeic-acid-treated cells confirmed apoptosis in dose- and time-dependent manner. Increasing ROS generation and reduction in the mitochondrial membrane potential were also accompanied in the caffeic acid-induced apoptosis. This work will promote caffeic acid as a likely candidate in the chemoprevention of colon cancer.

## 1. Introduction

Recent surveys indicated colorectal cancer as one of the most common and lethal cancers in Western states [1]. This cancer now paved entry into the Asian nations because of the people desire for Western diet which is growing rapidly. Although various conventional treatments exist, they still remain insufficient to eradicate this disease. Hence, the search for novel anticancer agents with better versatility in improving the disease condition is constantly increasing.

Previous work from our laboratory showed that honey could induce apoptosis in colon cancer cells. The apoptosis inducing potential of honey varied among the cell lines. On the basis of its total phenolic content, honey-induced apoptosis varied among the different honey types. Honey possessing higher phenolic content could inhibit the growth of colon cancer cells significantly [2, 3]. Hence, we extended our research to study the effect of caffeic acid, one of the most commonly reported phenolic constituent of honey, in colon cancer cells.

Caffeic acid is also widely found in coffee, fruits and vegetables [4]. It possesses both antioxidant and antitumor potential [5, 6]. Caffeic acid protected human KF1 diploid fibroblast and A431 epidermoid carcinoma cell lines from UVC-induced cytotoxicity [7]. It was shown to inhibit UVB (280–320 nm) radiation-induced IL-10 expression and the activation of the mitogen-activated protein kinases (MAPKs) in mouse skin [8]. In a recent work, it was shown caffeic acid inhibited the cell proliferation of HepG2 cells in a dose-dependent manner. It blocked the MMP-9 expression by inhibiting the NF-**κ**B activity. Finally, they showed that caffeic acid at a dose of 20 mg/kg retarded the growth of HepG2 tumor xenografts in immunosuppressive mice [9]. Caffeic acid was shown to inhibit the growth of HT 29 colon cancer cells. Caffeic acid at a concentration of 2500 *μ*M found to inhibit 50% cell proliferation of HT 29 cells [10]. However, no further work was initiated about the apoptosis induced by caffeic acid in colon cancer cells. Therefore, it is worthy to investigate the mechanism of caffeic-acid-induced apoptosis in colon cancer cells.

Programmed cell death or apoptosis is a major form of self destructive internally engineered cell death. It is complemented with diverse morphological changes like formation of membrane blebs, chromatin and nuclear condensation, segregation of apoptotic bodies, and DNA fragmentation. Reactive oxygen species (ROS) plays a pivotal role in various biochemical functions like cell proliferation and apoptosis. Many studies reported that ROS-mediated apoptosis is accompanied with the loss of mitochondrial membrane potential [11, 12].

In this present study, we aimed to examine the apoptotic nature of caffeic acid in colon cancer cells. Further we tried to explore the ROS and mitochondrial dependent mechanism in the apoptosis induced by the caffeic acid.

## 2. Materials and Methods

### 2.1. Reagents

RPMI 1640, fetal bovine serum (FBS), L-glutamine, sodium pyruvate, nonessential amino acids, vitamin solution, penicillin, and streptomycin were obtained from Life Technologies, Inc., Grand Island, NY, USA. 3-(4,5-dimethylthiazol-2-yl)-2,5-diphenyl-tertazolium-bromide (MTT), Propidium Iodide (PI), Rhodamine 123, RNase, and caffeic acid were purchased from Sigma-Aldrich, USA. YO-PRO-1 was obtained from Invitrogen Inc. USA.

### 2.2. Cell Culture

Colon carcinoma cell line HCT 15 (Organ: Colon, Disease: Colorectal adenocarcinoma; Organism: Human; procured from National Centre for Cell science (NCCS), Pune, India) was grown in RPMI medium supplemented with 10% FBS, L-glutamine, penicillin, sodium pyruvate, nonessential amino acids, and vitamin solution. Adherent monolayer cultures were maintained in T-25 flasks and incubated at 37°C in 5% carbon dioxide (CO_2_). The cultures were free of mycoplasma and maintained no longer than 12 weeks after recovery from frozen stocks.

### 2.3. Cell Proliferation Assay (MTT Assay)

Thiazolyl blue tetrazolium bromide (MTT) assay was carried out as follows. Cells were trypsinized and counted, and 1000 cells were seeded per well in 96-well plate. The following day, 100 *μ*L of medium containing the desired concentration of caffeic acid was added to the appropriate wells. The cells were then kept at 37°C in 5% CO_2_ for the desired length of time. Control used in these experiments was untreated cells kept for 48 h. For all the experiments performed below, control cells remained untreated and kept for the same duration as the longest time point of the respective experiment. At this point, 100 *μ*L of (5 mg/mL) MTT reagent was added to each well and the plate was placed at 37°C in the incubator for 2 h. 200 *μ*L of dimethyl sulfoxide was added to each well after aspirating the supernatant. Colored formazan product was assayed spectrophotometrically at 570 nm using ELISA plate reader [13].

### 2.4. Colony Forming Assay

HCT 15 cells were treated with caffeic acid (IC_50_ concentration estimated from MTT assay (800 *μ*M) was used for all experiments unless otherwise stated) for definite time periods (12, 24, and 48 h) and collected by trypsinization. The cells were counted and seeded again in triplicate on a 6-well tissue culture plate with 3000 cells/well. The cells were cultured for 15 days with growth media replaced after every two days. The cells were stained with 0.5% crystal violet (in methanol) and colonies were counted [14].

### 2.5. Relative Cellular Size and Granularity and Photomicrograph Images

Changes to the relative size and granularity of caffeic-acid-treated colon cancer cells were determined using flow cytometric analysis of forward and side laser light scatter characteristics. Further photomicrograph images of treated cells were acquired [13].

### 2.6. Cell-Cycle Analysis

After the appropriate treatment with caffeic acid, cells were washed with phosphate-buffered saline then resuspended in 50 *μ*g/mL propidium iodide containing 0.1% sodium citrate with 0.1% Triton X-100 for 20 min at 4°C. Cells were then analyzed by flow cytometry (FACScan; Becton Dickinson Immunocytometry Systems), and the sub-G_1_ fraction was used as a measure of the apoptotic cells. Control used in the experiments was untreated cells kept for 48 h. Analysis was performed in linear amplification mode in case of cell-cycle analysis. Remaining experiments of flow cytometry were performed in logarithmic amplification mode unless otherwise stated [13].

### 2.7. Estimation of ROS Generation

2′,7′-dichlorofluorescin diacetate (DCFH-DA) was cleaved by the intracellular nonspecific esterase to form DCFH. DCFH are oxidized by ROS to form the fluorescent compound DCF. Caffeic-acid-treated cells were harvested using Trypsin/EDTA and resuspended in PBS. Working solution (20 *μ*M) of DCFH-DA was directly added to cells, and then, it was incubated at 37°C for 15 minutes. Cells were washed and resuspended in PBS and kept on ice immediately before analyzing by flow cytometry [13]. This fluorescent intensity of DCF was measured and correlated with the ROS generated in the cells.

### 2.8. Determination of Mitochondrial Membrane Potential

HCT 15 colon cancer cells were treated with caffeic acid for different time points. Afterwards, cells were harvested and resuspended in 1 mL of rhodamine 123 (5 *μ*g/mL) for 1 h at 37°C. The intensity of fluorescence from rhodamine 123 was measured by flow cytometry [13].

### 2.9. YO-PRO-1 Staining

YO-PRO-1 iodide permits analysis of apoptotic cells without interfering cell viability. After treatment, the cell pellets were mixed in 1 *μ*M YO-PRO-1 for 20 min at room temperature before flow cytometric measurements [13].

### 2.10. Statistical Analyses

All values are expressed as the mean ± standard deviation. Figures were plotted with standard error using OriginPro 7.5 software.

## 3. Results

### 3.1. Cell Proliferation Assay

**Figure 1:**
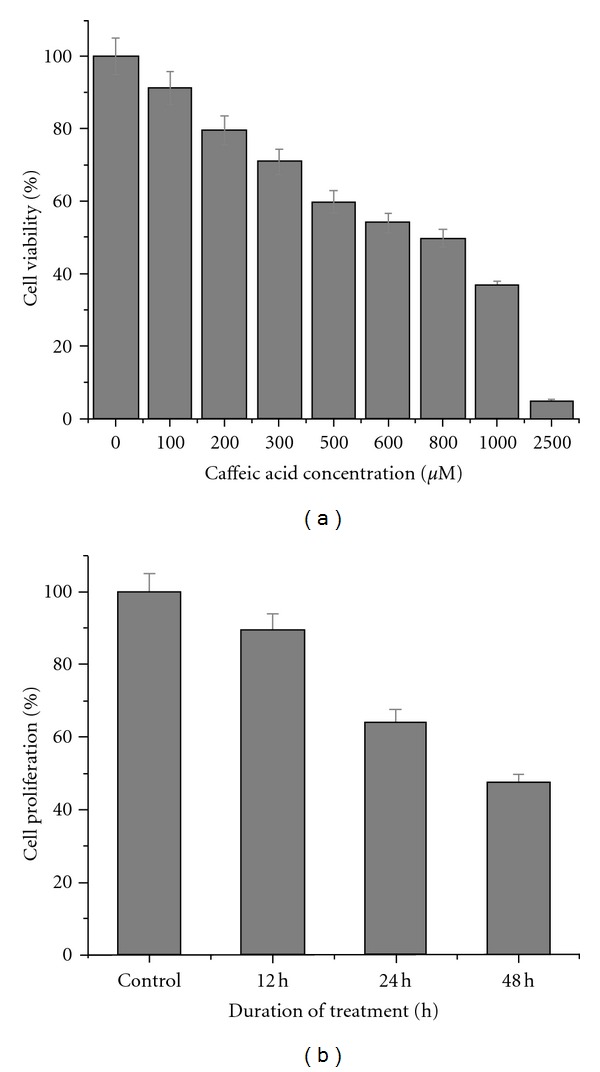
Antiproliferative effect of caffeic acid against colon cancer cells growth *in vitro. *(a) HCT 15 cells grown in 96-well plates were treated with various concentrations of caffeic acid diluted in the RPMI media for 48 h. The mean of the percentage cell viability (% of untreated HCT 15 cells) along with their standard error is indicated. (b) After various incubation periods of caffeic acid treatment, colonies formed were stained with 0.5% crystal violet and counted, and percentage of survival was calculated. Data reported as the mean ± SE from three different observations. Means are significant at 24 and 48 h (*P* < 0.05).

MTT assay was used to assess the antiproliferative effect of caffeic acid. Logarithmically grown HCT 15 cells were treated with varying concentration of caffeic acid for 48 h. Caffeic acid inhibited the growth of HCT 15 cells in a dose-dependent manner (Figure 1(a)). HCT 15 cell growth were repressed significantly with an IC_50_ (IC_50_: percentage at which 50% cells were dead approximately) of 800 *μ*M. We used IC_50_ concentration of caffeic acid for all our subsequent experiments unless stated otherwise.

### 3.2. Colony Forming Assay

Colony forming assay was used to assess the colony forming ability of caffeic-acid-treated cells. Untreated HCT 15 cells produced with an average mean of 95 colonies. Caffeic-acid-treated cells showed an average of 85, 61, 45 colonies after 12, 24, and 48 h of treatment. A time-dependent inhibition of colony formation was clearly evident from this experiment (Figure 1(b)).

### 3.3. Relative Cellular Size and Granularity and Photomicrograph Images

**Figure 2:**
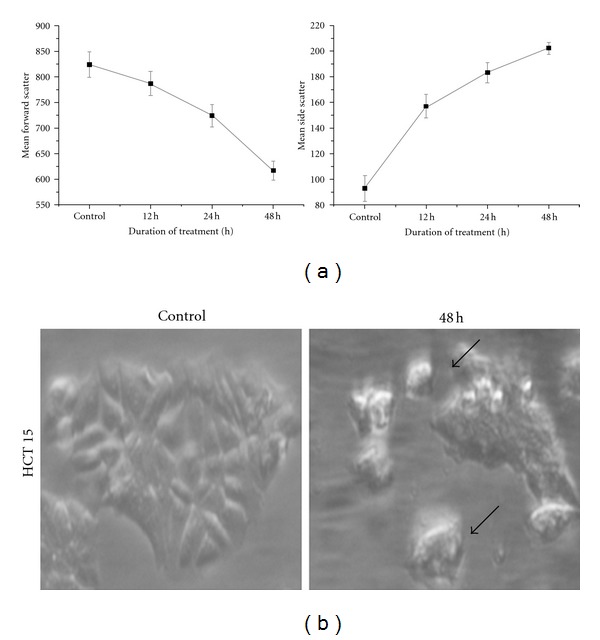
Changes to cell size and granularity distribution of caffeic-acid-treated cells. (a) Flow cytometric determination of cell forward and side laser light scatter was used to determine the relative changes to cellular size and granularity of HCT 15 cells after 12, 24, and 48 h of caffeic acid treatment. Data are mean ± S.E from the three independent experiments. (b) Photomicrograph of caffeic-acid-treated cells was acquired along with the untreated control cells after 48 h. Treated cells showed shrinkage compared to the normal cells as shown by arrow mark.

Generally apoptotic cells show a decreased relative size and increased internal complexity. Caffeic acid treated cells showed a decrease in the mean forward scatter and an increase in mean side scatter (Figure 2(a)) in a time-dependent manner while comparing with the untreated cells grown for 48 h. This may be attributed to the apoptosis induced by caffeic acid. Moreover, photomicrograph images of caffeic acid treated cells showed membrane blebbing and shrinkage of cells depicting apoptosis (Figure 2(b)).

### 3.4. Cell-Cycle Analysis

Cell-cycle analysis of caffeic-acid treated cells was performed using PI staining. Both dose- and time-dependent relationship of caffeic-acid-treated HCT 15 cells were examined. Dose-dependent analysis of 500, 1000 and 2000 *μ*M of caffeic acid showed an increasing sub-G_1_ arrest of 11.61, 28.27, and 33.10, respectively. Untreated control cells showed only 1.71% of sub-G_1_ after 48 h (Table 1). Similarly, we had performed time-dependent analysis of caffeic-acid-treated cells. It also showed an increasing sub-G_1_ arrest from 1.75% (control) to 25.27% after 48 h (Table 2).

### 3.5. ROS Generation

**Figure 3:**
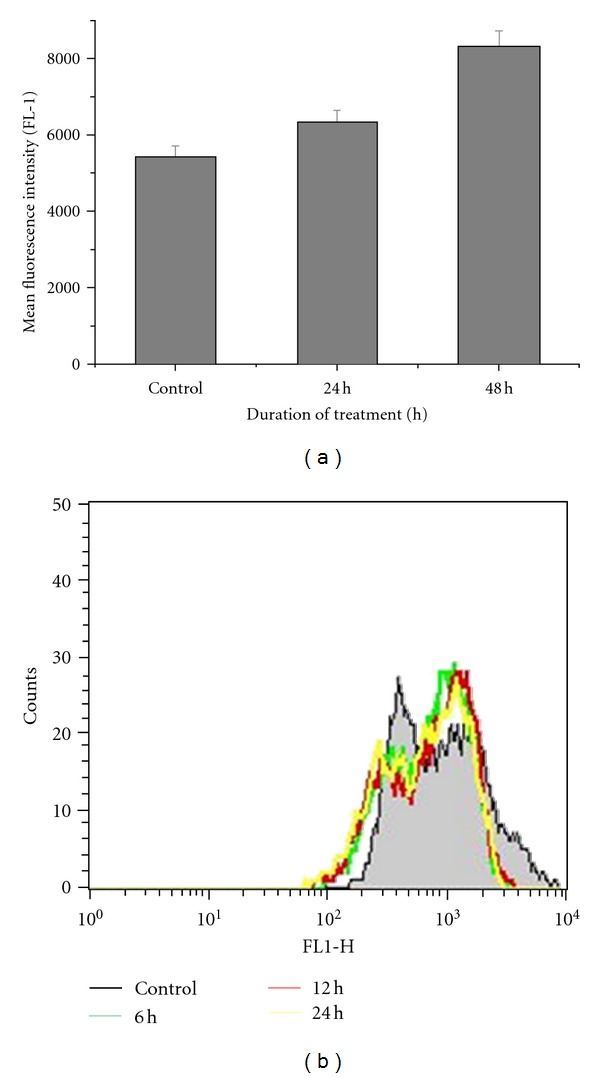
ROS generation and mitochondrial membrane potentials (Ψ_m_) of caffeic-acid-treated cells. (a) HCT 15 cells were cultured in the presence or absence of caffeic acid for the specified time points. DCFDH-DA fluorescence intensity was detected by using flow cytometry. Data shows the mean and standard error of three independent experiments. (b) HCT 15 cells were treated with caffeic acid for specified time-periods and then Ψ_m_ were determined using rhodamine 123 by flow cytometry. Data is representative of three independent experiments.

ROS generation is involved in the apoptosis of many anticancer agents. Here, we examined the alteration in the ROS levels of caffeic-acid-treated cells. We had found significant increase in the ROS levels after caffeic acid treatment. The increasing mean fluorescent intensity was found to be 6322, 8309 during 24 and 48 h, respectively. Untreated control cells showed an intensity of 5441 after 48 h (Figure 3(a)).

### 3.6. Mitochondrial Membrane Potential

Mitochondrial membrane potential was found to decrease in caffeic-acid-treated cells. The decreasing mean fluorescent intensity was found to be 834, 807, and 774 during 6, 12, and 24 h of treatment, respectively. Untreated control cells showed an intensity of 1155 after 24 h (Figure 3(b)).

### 3.7. Yo-Pro-1 Staining

**Figure 4:**
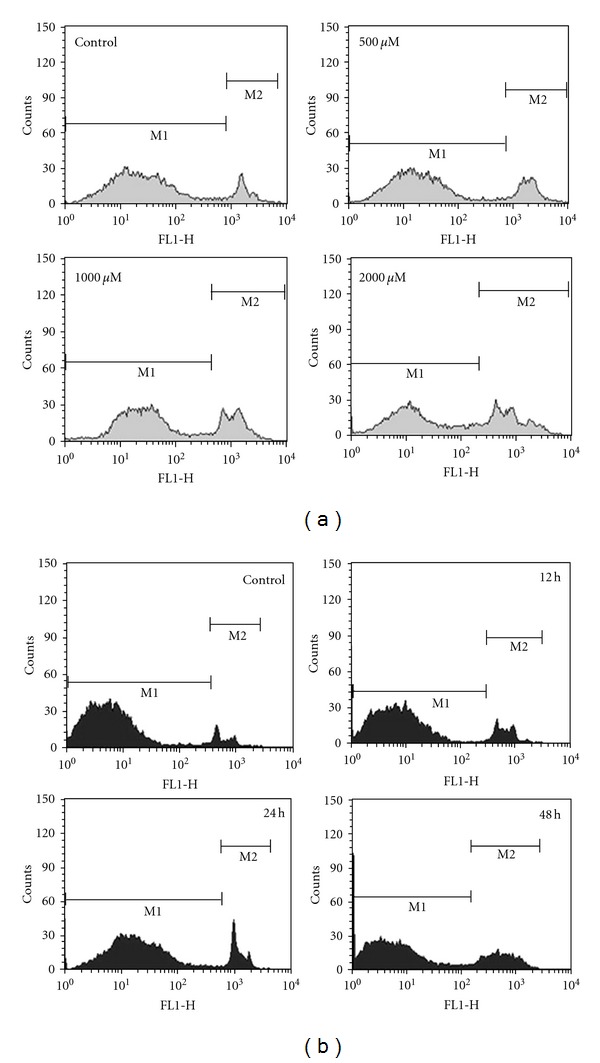
Apoptosis evaluation using Yo-Pro-1 dye by flow cytometry. (a) HCT 15 cells were treated with different doses of caffeic acid for 48 h. The distribution of cell population changed according to the doses as indicated by M1 and M2. Percentage of M2 population depicting apoptosis increased depending upon the increasing dose. Data is representative of three independent experiments. (b) HCT 15 cells were treated with caffeic acid for specified time points. The distribution of cell population changed according to the exposure time as indicated by M1 and M2. Percentage of M2 population depicting apoptosis increased on the basis of the duration of treatment. Data is representative of three independent experiments.

Apoptosis was assessed using YO-PRO-1 staining by flow cytometry. The percentage of cells distributed in M2 population signifying apoptosis increased depending upon the dose and duration of treatment. Dose-dependent analysis of 500, 1000, and 2000 *μ*M of caffeic acid showed an increasing M2 phase of 21.85, 30.06, and 39.01, respectively. Further, in time-dependent analysis, CA-treated cells showed increasing M2 phase population. It was found to be 13, 16.78, and 25.42 after 12, 24, and 48 h of caffeic acid treatment. M2 phase population of untreated control cells was found to be 8.62% after 48 h (Figure 4).

## 4. Discussion

Caffeic acid, one of the phenolic constituents of honey, significantly inhibited HCT 15 colon cancer cell proliferation. This result was in agreement with the previously published research which also showed inhibitory activity of caffeic acid against HT 29 colon cancer cells [10]. Colony-forming ability of cancer cells is essential for survival. Caffeic acid was found to inhibit the colony formation similar to some anticancer agent like Triphala (TPL), an Indian Ayurvedic formulation with known anticancer properties [14].

Selective killing of cancer cells using therapeutics is in constant demand for cancer. For this, targeting specific molecule using anticancer agents became the task, but problems like mutation and overexpression of the molecule make the task unfinished. Hence, better solution would be targeting specific biochemical reactions taking place inside the cell. One such thing is ROS, which plays a key role in various biochemical processes like apoptosis and cell proliferation. ROS stimulates the cell growth and proliferation hence increase in ROS is important in maintaining the cancer cell phenotype. However, high levels of ROS can also cause cellular damage, depending on the levels and duration of ROS stress [15, 16]. Hence, by exploiting these time- and dose-dependent ROS generation, we can trigger cell death by using exogenous ROS-generating agents. In our case, caffeic acid was found to increase the ROS generation in the colon cancer cells as shown by DCFDH-DA staining. Moreover, confocal images of DCFDH-DA staining also corroborated the increasing ROS generation in the caffeic-acid-treated cells (data not shown). Hence, caffeic acid may be overlooked as a potential exogenous candidate (generating ROS) to induce apoptosis in colon cancer cells.

Mitochondrial membrane potential also plays a major role in the regulation of physiological cell death (apoptosis) of animal cells [12]. There will be reduction in the cellular uptake of mitochondrial membrane potential sensitive dye like rhodamine123 while animal cells undergoing apoptosis. A fall in the Ψ_m_ will constitute an event in the apoptosis of many anticancer drugs [17, 18]. In caffeic-acid-induced apoptosis, we could evidence similar Ψ_m_ fall depicting that ROS-mediated apoptosis also accompanies mitochondrial dysfunction. This is greatly confirming with the results reported recently, where honey rich in phenolic content was also found to induce apoptosis by mitochondrial dysfunction [13]. Likewise, we hypothesize that the fall in the mitochondrial membrane potential and increased ROS generation may result in the activation of p53 in the caffeic-acid-treated cells. This may in turn, would have caused the upregulation of Bax and downregulation of Bcl_2_ which are the down-stream targets of p53 resulting in apoptosis. Hence, it would be appropriate to perform Western blot of the above-said proteins in the future research.

The hall mark of apoptosis is membrane blebbing and DNA fragmentation. Literatures stated that electron microscopic image is the golden standard for detecting apoptosis [19, 20]. Photomicrograph images clearly depicted the membrane blebbing and shrinkage. In this research, we showed that caffeic acid could arrest the cell cycle at the sub-G_1_ phase (an indicator of apoptosis) both in a dose- and time-dependent fashion using propidium iodide staining. This was similar to the effect of caffeic acid phenethyl esters (CAPEs) against C6 glioma cells [21]. Further, confocal images of the treated cells showed distinct DNA fragmentation (data not shown). Yo-pro-1 is widely used to detect apoptosis induced by anticancer agents [13]. Dose- and time-dependent staining by Yo-pro-1 demonstrated increasing accumulation of apoptotic cells after caffeic acid treatment. Induction of apoptosis was further substantiated by reduced forward and increased side scatter of the treated cells.

## 5. Conclusion

Caffeic acid, one of the phenolic constituents of honey, inhibited the colon cancer cell proliferation in a dose-dependent manner. Caffeic acid treatment resulted in increasing accumulation of cells at sub-G_1_ phase of cell cycle indicating apoptosis. Induction of apoptosis was accompanied by increased ROS generation as indicated by DCFDH-DA staining. Further mitochondrial membrane potential fall was also observed in the treated cells. Dose- and time-dependent staining by Yo-pro-1 demonstrated increasing accumulation of apoptotic cells after caffeic acid treatment. Photomicrograph images depicted the membrane blebbing and shrinkage of treated cells. Hence, caffeic acid can be considered as a potential candidate for inducing apoptosis in colon cancer cells through ROS and mitochondrial mediated mechanism. However, further experiments in preclinical and clinical settings will promote this as a likely candidate for chemoprevention of colon cancer.

## Figures and Tables

**Table 1:** Dose response of HCT 15 cells after caffeic acid treatment among various cell-cycle stages.

Dose (48 h)	Sub G_1_*	G_0_/G_1_	S	G_2_/M
Control	01.71 ± 3.10	27.31 ± 4.57	28.93 ± 1.71	39.72 ± 2.15
500 *μ*M	11.61 ± 2.78	19.81 ± 3.06	29.88 ± 2.58	37.66 ± 1.77
1000 *μ*M	26.27 ± 1.08	15.89 ± 1.98	20.45 ± 4.21	35.76 ± 2.32
2000 *μ*M	33.10 ± 2.23	14.27 ± 3.37	19.34 ± 1.30	30.70 ± 2.60

Data represents mean ± S.D; *means are significant at *P* < 0.05.

**Table 2:** Time response of HCT 15 cells after caffeic acid treatment among various cell-cycle stages.

Time in hours	Sub G_1_*	G_0_/G_1_	S	G_2_/M
Control (untreated, 48 h)	01.75 ± 2.75	26.84 ± 3.98	32.64 ± 1.23	36.51 ± 1.72
12 h	04.17 ± 1.83	38.90 ± 4.15	22.82 ± 3.29	32.57 ± 3.87
24 h	13.74 ± 1.52	25.04 ± 3.26	21.73 ± 1.78	38.13 ± 3.12
48 h	25.27 ± 3.01	19.54 ± 4.39	19.65 ± 3.76	33.71 ± 2.90

Data represents mean ± S.D; *means are significant at *P* < 0.05.
